# Supplementary figures and images for: Urban scaling of opioid analgesic sales in the United States
Source: PLoS One. 2021 Oct 12;16(10):e0258526. doi: 10.1371/journal.pone.0258526 (PMC8509933; doi:10.1371/journal.pone.0258526)

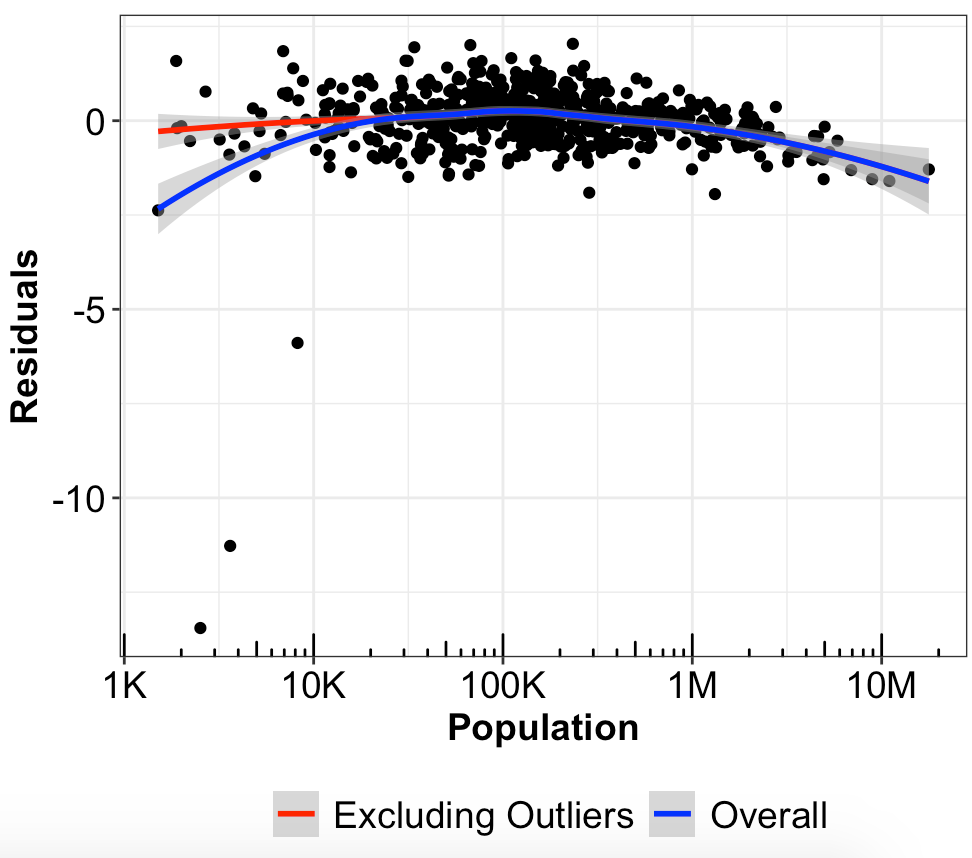

Supplement: S1 Fig — Source: ARCOS and Census Bureau*. *Copyright protection is not available for any work of the United States Government (Title 17 U.S.C., Section 105). Thus, you are free to reproduce census materials as you see fit. We would ask, however, that you cite the Census Bureau as the source. https://www2.census.gov/geo/pdfs/maps-data/data/tiger/tgrshp2019/TGRSHP2019_TechDoc.pdf. Footnote: blue line is a loess smoother of standardized residuals on log(population) including all commuting zones; the red line excludes the three strong outliers. (TIF) [file pone.0258526.s001.tif]

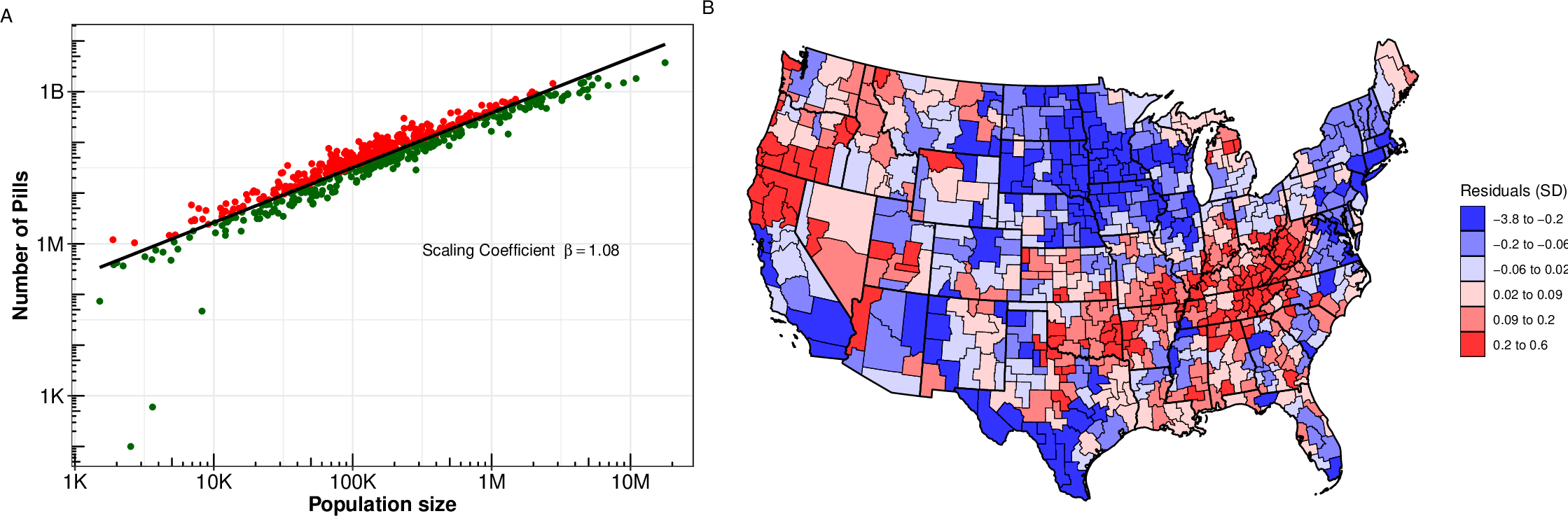

Supplement: S2 Fig — Footnote: β is the coefficient of the regression log(pills) on log(population). Red-colored CZs represent positive residuals and green-colored CZs represent negative residuals. Source: ARCOS (through the Washington Post) and Census Bureau. (TIF) [file pone.0258526.s002.tif]

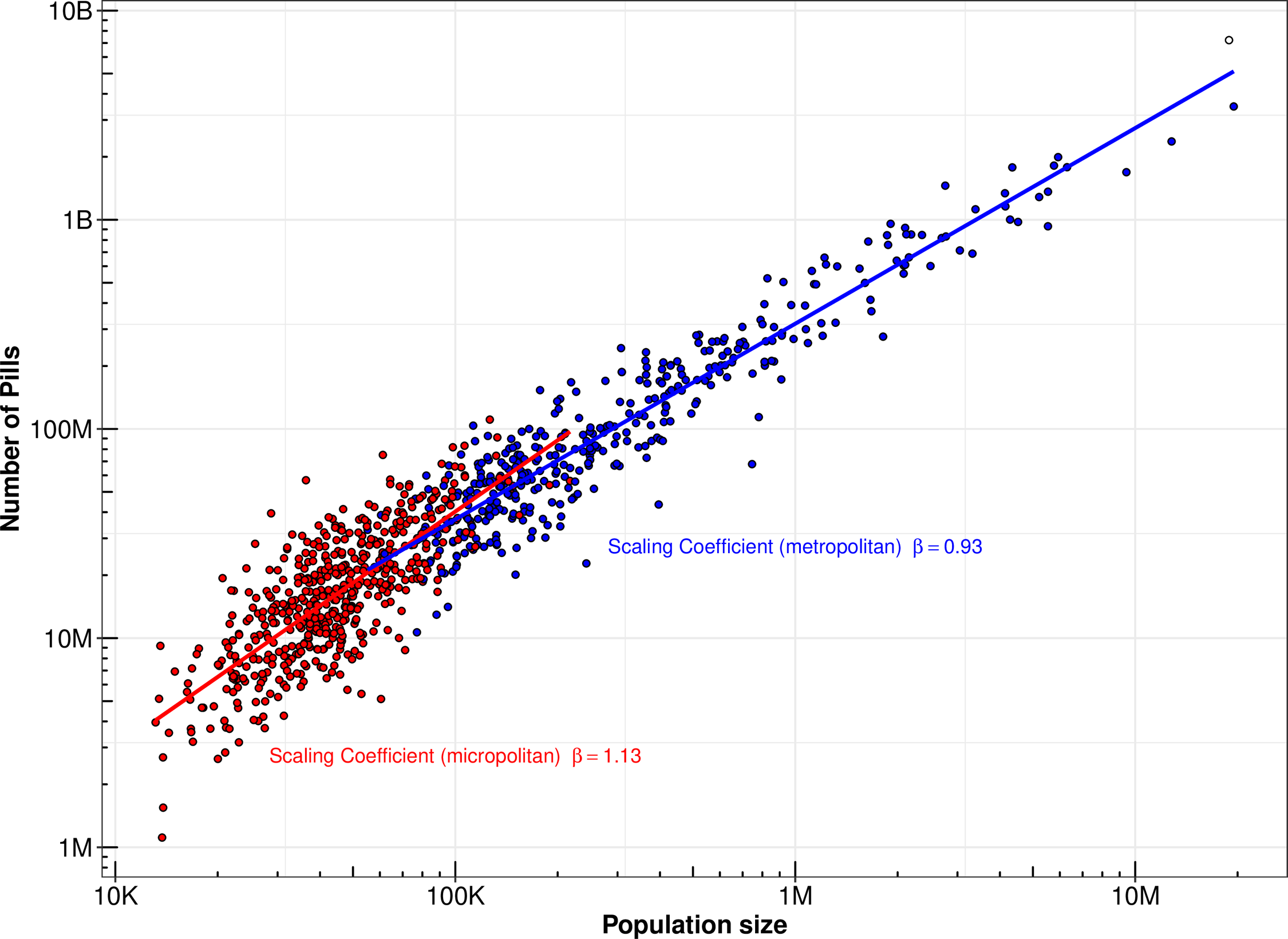

Supplement: S3 Fig — Footnote: β is the coefficient of the regression log(pills) on log(population). Micropolitan CBSAs (red) are those built around an urban cluster with population between 10,000 and less than 50,000 people. Metropolitan CBSAs (blue) are those built around urban clusters of 50,000 people or more. Sources: ARCOS and Census Bureau. (TIF) [file pone.0258526.s003.tif]
