# Supplementary material for: Urban scaling of opioid analgesic sales in the United States
Source: PLoS One. 2021 Oct 12;16(10):e0258526. doi: 10.1371/journal.pone.0258526 (PMC8509933; doi:10.1371/journal.pone.0258526)
Supplement: S1 Table — (DOCX) [file pone.0258526.s004.docx]

**S1 Table. Scaling coefficients from adjusted models compared to unadjusted models.**

|  | n | β_1_ (95% CI)^a^ | β_2_ (95% CI)^a^ |
| --- | --- | --- | --- |
| Unadjusted | 607 | 1.36 (1.23-1.50) | 0.92 (0.88-0.95) |
| Adjusted for age categories | 607 | 1.42 (1.27-1.57) | 0.94 (0.91-0.98) |
| Excluding outliers^b^ | 604 | 1.14 (1.07-1.21) | 0.92 (0.88-0.95) |
| Excluding outliers^c^ | 604 | 1.15 (1.10-1.21) | 0.91 (0.87-0.95) |

^a^β_1_ and β_2_ are the scaling coefficients below and above the knot (82,363)

^b^ Excluding outliers but keeping the same knot (82,363)

^c^ Excluding outliers and re-calculating the optimal knot (151,631)
