# Supplementary material for: Urban scaling of opioid analgesic sales in the United States
Source: PLoS One. 2021 Oct 12;16(10):e0258526. doi: 10.1371/journal.pone.0258526 (PMC8509933; doi:10.1371/journal.pone.0258526)
Supplement: Attachment — Submitted filename: Response to reviewers.docx [file pone.0258526.s005.docx]

**Response to reviewers**

Manuscript: Urban scaling of opioid analgesic sales in the United States

We thank the editor and the reviewers for their comments. We appreciate your time and are confident that this version of our manuscript is significantly superior to the original version submitted. Please see below a point-by-point response to the comments.

| Comment | Response |
| --- | --- |
| **Editor** |  |
| It is an important topic, and the results would deserve to have a nice publication. However, the paper misses in my point of view the theoretical hypotheses that would strengthen the interpretation. In particular, it does not contextualize the relation between medical treatments / deseases / medical infrastructure. It is argued that there are no studies on scaling and health that is not true: see for example L. E. C. Rocha, A. E. Thorson, R. Lambiotte (2015). The non-linear health consequences of living in larger cities, Journal of Urban Health 92 (5) and some others since that publication.... so please highly strength the state of the art and the reflexion (fllowing also reviewer 1) | Thank you for your comments. We have now added information to clarify the mechanisms underlying the scaling phenomenon of various outcomes.  In the case of health outcomes, we also clarified the relationships between access to medical resources and the scaling behaviors of health outcomes. We also included other studies examining the scaling of health outcomes, including Rocha et al. (pages 4-5) See version with track changes |
| **Reviewer 1** |  |
| (a)The submission tackles a very interesting question but fails in several ways. (a) The choice of spatial unit is not sufficiently justified. | Thank you for your comments.  We have now added a justification for the use of commuting zones (page 5, paragraph 2, and page 12). See version with track changes. |
| (b) Scaling is not about size but about how interactions are affected by scale. Thus one would not expect similar scaling behavior across spatial units which might represent different interactions. | Thank you for pointing this out.  We have now added information to clarify the mechanisms underlying the scaling phenomenon of various outcomes (pages 3-5).  In the case of health outcomes, we also clarified the relationships between access to medical resources and scaling behaviors of some outcomes. We also included other studies examining the scaling of health outcomes. (pages 4-5)  We agree that the use of different spatial units can affect the scaling behavior. We have indicated that in the limitation section (page 12). To test whether our choice of spatial unit had an influence on the scaling behavior, we replicated the analysis using a different spatial definition– the Core-based Statistical Areas (CBSA). (S1 Fig 3). This analysis showed consistent result with those obtained from commuting zones. |
| (c) The methodological choice of a linear spline at the population mean is not justified. | We have now added the justification for this choice in the method section (page 6-7) |
| **Reviewer 2** |  |
| The manuscript is technically sound. The content is informative, new and interesting to public health institutions and also individuals seen the huge problematics of opioide misuse in the US. 2. I am no statistics expert, so I can not really evaluate the statistics topic. 3. Neverthesless from my limited understanding of statistics the data seem to be complete. 4. The manuscript is understandable and written in standard English (I found only some small typos). | Thank you for your comments.  We have carefully reviewed the manuscript and fixed these typos. |
| **Other comments regarding format/data availability** |  |
| Please ensure that your manuscript meets PLOS ONE's style requirements, including those for file naming. | We have now updated the manuscript according to PLOS ONE style requirements. |
| We note that you have included the phrase “data not shown” in your manuscript. Unfortunately, this does not meet our data sharing requirements. PLOS does not permit references to inaccessible data. We require that authors provide all relevant data within the paper, Supporting Information files, or in an acceptable, public repository. | We have included a table in the supporting information containing the result previously described as “data not shown”. We have also updated the text to refer to this table (S1 Table) on page 8, paragraph 1. See version with track changes. |
| We note that [Appendix 1 and Figure 2] in your submission contain [map/satellite] images which may be copyrighted. All PLOS content is published under the Creative Commons Attribution License (CC BY 4.0), which means that the manuscript, images, and Supporting Information files will be freely available online, and any third party is permitted to access, download, copy, distribute, and use these materials in any way, even commercially, with proper attribution. | We have not used any copyrighted material. The maps included in the manuscript were created by us using census bureau cartography boundary files. We cited the Census Bureau as a source.  We uploaded the information from the Census:  Copyright protection is not available for any work of the United States Government (Title 17 U.S.C., Section 105). Thus, you are free to reproduce census materials as you see fit. We would ask, however, that you cite the Census Bureau as the source.  https://www2.census.gov/geo/pdfs/maps-data/data/tiger/tgrshp2019/TGRSHP2019_TechDoc.pdf |
| Please include captions for your Supporting Information files at the end of your manuscript, and update any in-text citations to match accordingly. | We have now done that. |
| The [PLOS Data policy](https://nam01.safelinks.protection.outlook.com/?url=http%3A%2F%2Fwww.plosone.org%2Fstatic%2Fpolicies.action%23sharing&data=04%7C01%7Cphm32%40drexel.edu%7Cea94394f27a148c5686708d874294481%7C3664e6fa47bd45a696708c4f080f8ca6%7C0%7C0%7C637387067873717286%7CUnknown%7CTWFpbGZsb3d8eyJWIjoiMC4wLjAwMDAiLCJQIjoiV2luMzIiLCJBTiI6Ik1haWwiLCJXVCI6Mn0%3D%7C1000&sdata=RQCEGC0hG7LhJPDYrnWhQqGgV2CAFKngD2fg4P2t8VQ%3D&reserved=0) requires authors to make all data underlying the findings described in their manuscript fully available without restriction, with rare exception (please refer to the Data Availability Statement in the manuscript PDF file). The data should be provided as part of the manuscript or its supporting information, or deposited to a public repository. | We will be making the data and code available in a public repository: https://github.com/usamabilal/ARCOS_Pill_Scaling/  We also included the link in the manuscript (page 6, paragraph 1). |
