# Supplementary material for: Urban scaling of opioid analgesic sales in the United States
Source: PLoS One. 2021 Oct 12;16(10):e0258526. doi: 10.1371/journal.pone.0258526 (PMC8509933; doi:10.1371/journal.pone.0258526)
Supplement: Attachment — Submitted filename: Response to reviewers.docx [file pone.0258526.s006.docx]

**Response to reviewers.**

Thank you for your comments. Below we provide a point-by-point response to the comments.

| Reviewer #2: It seems that there has been a substantial improvement in content and, as mentioned by reviewer 1, clearing of other available contextual literature and research. It also seems that all data are fully available and accessible on the gitHub platforme. In the abstract there is still a small typo (line 14: superlinear (not superliner). Soures / literature are added and also explained. The structure of the document is now much clearer. (All this under the precondition that I am not an expert in statistics neither in opioid/medical-social topics). | Thank you so much for your careful review of our manuscript. We have fixed the typo indicated. |
| --- | --- |
| Reviewer #3: Report on “Urban scaling of opioid analgesic sales in the United States”  The authors analyze the prescription of opioid pills in commuting zones (CZ) in the USA. Analyzing the entire set of CZ they find super-linear scaling, ie in large cities more pills are prescribed. However, the authors find that the residuals exhibit a systematic deviation in a U-shape from which the authors infer two different scaling regimes. Separating the CZ into two groups according to the median, a super-linear (below the median) and a super-linear (above the median) regime are found. The authors hypothesize reasons for this different scaling regimes.  Overall, this is a nice little paper. It is mostly well written and relevant to the urban scaling community and probably also for the opioid-crisis community. Accordingly, I recommend publication.  The only (non-mandatory) thing that the authors could consider is a better statistical treatment. They could automatically find a best division value for the two regimes (instead of the median), ie an optimization. In addition, colleagues with statistics background would appreciate seeing some test statistics that the model with two regimes fits better than the model with only one beta. In this context Akaike Information Criterion might be useful. | Thank you so much for your careful review.  We agree with the reviewer and have re-run the analysis with the spline using a data-driven approach. Specifically, we have now explored for the best-fitting spline knot, selected as the knot that maximizes model fit. While results are not substantially different from our original findings, we have now reported this as the main analysis. We have also provided a measure of fit (AIC) between the no-spline model and the model with a spline, finding a much better fit in the spline model.  We have edited the methods section, paragraph 4, and throughout the results section.  results section page XXX, and results section. |
| **Specific comments:** |  |
| - which location is use, address of patient, doctor, or pharmacy? | We used the location of the pharmacy or clinic. We have added sentences to make that clean in the text on pages 5 (last paragraph) and 6 (paragraph 2). See version with track changes. |
| - “Scaling is the response of complex systems, such as cities, to changes in their size.” might be misleading. In most cases urban scaling is studied cross-sectionally (fixed year), but “changes” suggests change over time | The have changed to “variations” in size. |
| - beginning of page.11: “For example” is just repeating what is already said in the previous sentence | We have now removed this sentence as it was, as indicated by the reviewer, completely redundant. See paragraph 3 of the introduction. |
| - “disporportionally high number of social connections in large cities leads to an exponential increase in various outcomes such as economic productivity and number of patents”: the increase is probably not exponential | We meant to say: ”high number of social connections in large cities leads to a disproportional increase in various outcomes…” Thank you for identifying this issue. We have now fixed it. See track changes in paragraph 3 of the introduction. |
| - why 607 CZ? Is this the total number? If not, how have they been chosen, why have others been omitted? | 607 are all CZs in the continental US. We have clarified that and explained the rationale in the first paragraph of the methods. |
| - “After visually exploring initial results, we detected a strong non-linear pattern” In log-log representation, I assume. | Yes. We have clarified that in the fourth paragraph of the method section. |
| - how do the authors deal with zero-values? Ie are there any CZ with no pills? If they, then the log-value cannot be shown | All CZs had at least one pill so there were no CZs with zero values. The number of CZs (607) in the final model can be seen on the tables. We have also added that information in the title of Figure 1. |
| - why are the Figures in the SI? | SI (Supplementary Information) contain the figures and tables that resulted from the sensitivity or exploratory analysis that were not part of the main analysis.  Regarding the main paper figures, it may be that PLOS ONE is grouping them after the SI in the review PDF. |
| - “we added a linear fit with a linear spline at the population median” spline is not visible, is the linear regressions are discontinuous | The linear regressions are discontinuous because they represent two different slopes: one for the CZs above the median and the other for the CZs below the median. However, to make this clearer, we have added a flag on the main figure indicating where the knot position is. |
| - Tab.1: it is not clear how adjustment and stratification has been done | We have added information about the adjustment and region-specific analysis on page 7 (paragraph 2). See version with track changes. For the adjustment we used dummy variables for regions. For the stratified analysis, we estimated coefficients for each region separately. We have also added a footnote to table 1. |
| - somehow the authors describe more Figures than were in my pdf | We are sorry about this. This issue may emerge from some figures that are in the supplementary information. We have now corrected this. We have also re-ordered the description of the Figures and Tables in the result section to prioritize results from the main analysis followed by results from the sensitivity analysis (which were included in the SI). |
| - how do the years 2006-2014 go into the analysis? Is it that the values simply represent the total pills prescribed in this period? | We summed all pills for the period for each CZ. We have now added this information in the third paragraph of the methods section. |
