# Supplementary material for: Urban scaling of opioid analgesic sales in the United States
Source: PLoS One. 2021 Oct 12;16(10):e0258526. doi: 10.1371/journal.pone.0258526 (PMC8509933; doi:10.1371/journal.pone.0258526)
Supplement: Attachment — Submitted filename: Response to reviewers.docx [file pone.0258526.s007.docx]

**Response to reviewers**

Thank you for your comments. Below we provide a point-by-point response to the comments.

**Reviewer #2:** It is visible see that you worked hard on the last version and answered to all comments of the reviewers. It seems to me (as already mentioned, I am not a statistics expert) that you did a lot of new research.

I found the discussion and explanations with the reviewer very interesting and helpful.

Maybe in general it is helpful to explain results in this way. It depends also on the target group how you formulate your text. Myself as a scientist with a backgroud in Geology/Paleontogy and Systems Scientist I am always working on making results easily understandable (also for "oursiders").
Therefore I very much appreciate your documentation of the discussion and review process.

Finally two small typos again in the downloaded Fig 2 & 3: I suppose the legend should be named "coefficient" (not "coefificient").

**R:** Thank you for your comments. We appreciate the time and effort put in this review. We genuinely believe that this has made our paper much stronger.
We have now fixed the typos in Fig 1 and supplemental Figs 2 and 3. Thank you.
