# Supplementary material for: Urban scaling of opioid analgesic sales in the United States
Source: PLoS One. 2021 Oct 12;16(10):e0258526. doi: 10.1371/journal.pone.0258526 (PMC8509933; doi:10.1371/journal.pone.0258526)
Supplement: Attachment — Submitted filename: Response to reviewers.docx [file pone.0258526.s008.docx]

**Response to reviewers**

Thank you for your comments. Below we provide a point-by-point response.

**Reviewer #4: 1**. It would help to better justify the research approach. All prescription opioid counts from 2006 to 2014 were aggregated for each county over a period of increasing prescriptions. Why isn’t the outcome of interest the growth of prescriptions over the time period rather than the total amount? (It seems like from a policy perspective, you would want to know where prescriptions increased substantially given the introduction section.) Do any of the CZs grow substantially over the time period? If so, then this seems like information you would want to examine (if not, it justifies the averaging of population size and should be stated). That is, do fast growing populations correlate with fast growing prescriptions? Is this a confounder to worry about? What I’m getting at is the paper reads as if a couple variables were chosen without much thought to see if they were or weren’t correlated. Why is the chosen correlation the one to focus on rather than a different one? Explaining this would help justify the statistical approach.

**Response:**

Thank you for your comments. The growth in opioid prescription has been described elsewhere in the vast literature about the US opioid epidemic (see for example Jalal Science 2018, describing a long-term exponential growth in opioid deaths). Instead, the aim of our paper was to understand how opioid prescriptions scale with city size by leveraging the urban scaling framework. From the perspective of the population size, population did not change considerably in the period studied (median relative change from 2006 to 2014=+2.3% [IQR: -0.2% to 5.4%]), playing into our decision to use the pooled period rather than the year-by-year trend. Population growth can also be an important driver of health outcomes. This issue is being examined in other papers we are currently working on, but the examination of this phenomenon extends beyond the aim of this paper.

The variable population size was chosen after much thought considering the urban scaling framework. Scaling is the response of complex systems, such as cities, to variation in their size. The application of this framework has previously shown that a set of scaling relations can be used to predict several features of cities. Specifically, for the outcome opioid pills, one possible mechanism explaining the relationship between population size and opioid pills is that a disproportionally large number of social connections in relatively larger CZs creates an environment that facilitates a larger than expected number of successful matches between prescribers and patients, which in turn may lead to superlinear scaling of opioid analgesic pills. We explain these relationships in detail in the introduction and discussion of the paper. We have now made several edits to the introduction to make the aim and the theoretical framework more explicit.

**2.** It’s not clear why Alaska and Hawaii were excluded. Are they weird outliers for some reason? At a minimum the authors should explain more clearly why they should be excluded and test whether the estimates change in any meaningful way if Alaska and Hawaii are included. If there is no real difference, this should be stated. If the estimates do change in meaningful ways, then omitting them really needs a stronger justification.

**Response:**

We excluded Alaska and Hawaii because the commuting patterns in these states are expected to be different from those in the continental US since they are not connected by land to other states. Commuting patterns are key in the definition of Commuting Zones (CZs), one of the geographic delimitations we used in this paper. We have the justification in the text of the article. “We excluded CZs that include counties in non-contiguous states (Alaska and Hawaii) because they may not be a good representation of these networks that, in the continental US, often cross state lines.”

**3.** The U-shaped pattern in Fig S1 seems largely driven by the three outlier residuals in the lower left of the figure. Indeed, when omitted in Table S1, the coefficient for beta1 falls a large amount. Although the “findings are robust” to this, meaning the signs of the coefficients and statistical significance presumably don’t change, the magnitude of the coefficient changes and needs to be discussed. Does the U-shape disappear? Does the optimal spline change? Are the outliers located in a particular region, perhaps explaining some of the stratification results?

**Response:**

We thank the reviewer for this important insight. These three outliers are three small commuting zones (<10,000 pop) in New Mexico, South Dakota, and Montana. We have modified Fig S1 to reflect how the u-shape changes after removing these outliers. The pattern above for larger cities remains unchanged, but the pattern for smaller cities is now flat. This indicates that while the model without a spline is a good fit to smaller commuting zones, it still fails to reproduce patterns of opioid prescribing in larger cities. We have now reflected this in the text and in Figure S1. We have also explored whether the spline knot changes, and indeed it does change from 81,000 to 151,000 (very close to the median of 154,000). We have now added an extra robustness check by showing the scaling coefficients without the outliers and with the new threshold. As with the previous sensitivity analysis, the direction (and significance) of coefficients remains unchanged, although there’s a weaker superlinearity in smaller commuting zones (going from 1.36 in the main analysis to 1.24 after excluding outliers and changing the knot location).

4. In Table 1, what does “adjusted for region” mean? Does it mean a dummy variable was included for each region in the estimates? Or something else? This can be clarified in the text or Table 1 notes.

**Response:**

We have added a footnote in Table 1 indicating that we included dummy variables for region (or stratified, in the case of stratified models):

“^b^ Models adjusted for region included dummy variables for each region.

^c^ Stratified models included the CZs for each region separately.”

5. There is no interpretation of the coefficients. What does a beta1 of 1.42 vs 1.17 mean from a practical perspective? Both are superlinear, but does the difference of magnitude mean anything? How should they be interpreted? Why should we care about these specific numbers? Does a beta1 of 1.01 and a beta2 of 0.99 have any real meaning? If not, what level of these coefficients should matter? Or does it not matter and only the superlinear or not question is what matters (if so why?)?

**Response:**

We have now added the interpretation to the results. See results:

“The scaling coefficient for opioid analgesic pills in all 607 US Commuting Zones from 2006 to 2014 was 1.08 (95% CI 1.05-1.11), corresponding to superlinear scaling. These results show that the number of analgesic opioid pills was disproportionately higher in large (vs. small) CZs (S2 Fig.). Specifically, a CZ with 1% larger population had 1.08% greater pill count. However, we found that the model introducing a spline had a better fit than the model without a spline (AIC=127.8 in the model with a spline vs AIC=191.3 in the model without a spline), indicating a non-linear scaling behavior. Fig. 1 shows that CZs with population below the knot (population of 82,363) scale superlinearly (β=1.36, 95%CI 1.23 to 1.50), and CZs with population above the knot scale sublinearly (β=0.92, 95%CI 0.88 to 0.95). This means that for CZs below the knot, a 1% larger CZ had a 1.36% higher pill count, while for CZs above the knot, a 1% larger CZ had a 0.92% higher pill count.”

A superlinear scaling coefficient of 1.08 means that a CZ with a 1% larger population had a 1.08% greater pill count, meaning that that the pill count was disproportionally larger even after accounting for the fact that the CZ has greater population. A larger coefficient (or smaller in the case of sublinear scaling) means stronger scaling which is important to characterize the behavior of cities regarding specific outcomes.

**6.** If it is really a U-shaped relationship, why not use a quadratic estimation equation (add a population squared term)? Does it fit the data better? If so, how would you interpret the results?

We agree with the author that a quadratic polynomial for population (so log(pills)=b0+b1*logpopulation+b2*logpopulation^2) would also probably fit the data best. However, interpreting such coefficients is challenging, as they would no longer provide an estimate of the scaling behavior of opioid pill sales. Our approach is a version of the “power law with cut-off” approach described in Clauset et al. (SIAM 2009). We have now referenced this on the methods section.

**7.** The discussion needs work. The first sentence of the second paragraph is “Our results highlight the importance of exploring nonlinear scaling”, but nowhere is the “importance” explained. That it is detected in the estimates does not mean it is “important”. More care needs to be taken in explaining the importance of the results if this is the goal. (PLOS ONE does not judge things based on importance, so the highlighting of the importance could be removed if the author does not want to justify the importance of the results.)

**Response:**

We adjusted the discussion to address this issue by not mentioning the importance of exploring nonlinear scaling. This paragraphs now reads: “Our results show the nonlinear scaling behaviour of opioid pills, a pattern that has been found in other outcomes”. The goal of the paper was to explore the distribution of pills using the urban scaling framework. We believe this can be important to understand larger patterns in cities.

**8.** Discussion says “One potential explanation [that below a threshold, higher populations are more strongly correlated with more opioid prescriptions, and above it, less] is that increases in potential matches beyond a certain threshold no longer translate into higher rates of successful matches.” But isn’t it that beyond some threshold, the higher rates of successful matches slows down, not that it disappears? Or am I missing something? It’s not like there is a sharp break at a threshold, but a (very) gradual flattening of the curve.

**Response:**

Thank you for identifying this issue. We have adjusted the discussion. It now says: “One potential explanation is that the rate of successful matches decreases beyond a certain threshold”.

**9.** Several potential explanations are given in the discussion, but it would be helpful if it was explained why these (or other confounders) were not included in the analysis. Can you control for number of pharmacies/prescribers or not? Levels of prescription opioid vs heroin deaths in a CZ? It seems there are a potential long list of confounders that the limitations should highlight (poverty rates, unemployment, demographic profile, number of physicians, income level, existence of PDMP programs, opioid treatment availability, etc.). In addition, since PLOS ONE requires "appropriate controls", the lack of any controls needs justification.
**Response:**

Variables such as number of pharmacies, number of physicians, and poverty are hypothesized to be part of the mechanism for why population size is associated with opioid pills. Adjusting for these variables would block the mechanisms that lead to the association. For example, a larger population may lead to a larger concentration of physicians which in turn leads to a larger number of prescriptions. Given that we want to examine the broader relationship between population and outcome and describe the world as is, we believe that these variables should not be adjusted for. Future studies looking to explain mechanisms behind these patterns can explore whether scaling behaviors are attenuated after controlling for these variables. We have added this justification in the method section: “We used the model described above following standard practice in the urban scaling literature and did not adjust for any variables that may be in the pathway between population size and the outcome.”. We have also indicated in the discussion that future studies may want to explore these mechanisms.

Small typo: “42th” is used instead of 42nd.

**Response:** Thank you. We have now fixed that.

**Reviewer #5**: The authors did a great job addressing previous reviewers comments. The revisions helped to clarify the methods and results.
On page 5, line 6, "maybe" should be "may be"
On page 10, line 7, "Last" should be "Lastly"
On page 11, line 3, insert "be" between "could" and "the"
On page 12, line 12, insert a reference for the statement about patients filling prescriptions close to home or work.

**Response:**

Thank you for your additional comments. We have now fixed the issues that you raised. We have re-phrased the sentence on page 12, line 12, to reflect a general statement about commuting zones (vs. a specific statement about where patients fill their prescriptions, for which we do not have data).
